# Rare Association of Anti-Hu Antibody Positive Paraneoplastic Neurological Syndrome and Transitional Cell Bladder Carcinoma

**DOI:** 10.1155/2012/724940

**Published:** 2012-12-20

**Authors:** S. Lukacs, N. Szabo, S. Woodhams

**Affiliations:** ^1^St Mary's Hospital, Imperial NHS Healthcare Trust, Praed Street, City of Westminster, London W2 1NY, UK; ^2^King's College Hospital NHS Foundation Trust, London SE5 9RS, UK; ^3^Department of Urology, Western Sussex Hospitals NHS Trust, Worthing BN11 2DH, UK

## Abstract

*Introduction*. Paraneoplastic encephalomyelitis (PEM) and subacute sensory neuronopathy (SSN) are remote effects of cancer, usually associated with small-cell lung carcinoma and positive anti-Hu antibody. We describe the rare association of bladder transitional cell carcinoma (TCC) with anti-Hu antibody positivity resulting in this paraneoplastic neurological syndrome. *Patient*. A 76-year-old female presented with bilateral muscle weakness and paraesthesia of the upper and lower limbs in a length-dependent “glove and stocking” distribution. Central nervous system symptoms included cognitive problems, personality change, and truncal ataxia. Case notes and the literature were reviewed. *Result*. Autoantibody screening was positive for anti-Hu antibody (recently renamed antineuronal nuclear antibody 1, ANNA-1). The diagnosis of PEM and SSN was supported by MRI and lumbar puncture results. A superficial bladder TCC was demonstrated on CT and subsequently confirmed on histology. No other primary neoplasm was found on full-body imaging. The neurological symptoms were considered to be an antibody-mediated paraneoplastic neurological syndrome and improved after resection of the tumour. *Discussion*. The association of anti-Hu positive paraneoplastic neurological syndrome and TCC has not been described in the literature previously. We emphasize the need for detailed clinical examination and the importance of a multidisciplinary thought process and encourage further awareness of this rare association.

## 1. Introduction

 The antineuronal nuclear antibody 1 (ANNA-1) previously called as anti-Hu antibody directed against intracellular antigens is a polyclonal IgG (35–40 kD) type antibody which binds to tumours and neural cells [1]. The binding can cause neurological symptoms such as sensory neuropathy, cerebellar ataxia, limbic encephalitis, brainstem encephalitis, intestinal pseudoobstruction, parietal encephalitis, or multifocal involvement as part of a paraneoplastic neurological syndrome [2]. These symptoms usually precede the diagnosis of the primary cancer, which is most of the time small in size and is found in an early, nonmetastatic phase. Most of these tumours are small-cell lung carcinomas (SCLCs) but there are other rare associations with ovarian, breast, prostate, cervical cancer, thymoma, and Hodgkin's lymphoma [3–6]. A thorough review of the literature found no reported association between anti-Hu positive paraneoplastic neurological syndrome and transitional cell carcinoma (TCC) of the bladder.

 TCC of the bladder is the second most common urological malignancy. Known risk factors are male gender (3-4-fold), old age with a peak at the 8th decade, tobacco smoking (4-aminobiphenyl, 2-naphthylamine), occupational exposure to carcinogens (in particular aromatic hydrocarbons i.e., aniline), certain drugs (i.e., cyclophosphamide, phenacetin), white race, environmental carcinogens and, pelvic irradiation [7]. The WHO histological classification from 1973 differentiates 3 groups of bladder cancer, such as well (G1), moderately (G2), and poorly differentiated (G3) bladder cancer. TCC can be single or multifocal. 1.7%–5% of the patients have synchronous upper tract TCC, while metachronous recurrence can also develop several years after the initial diagnosis [8]. 

In this paper we present a female patient with anti-Hu antibody who had presented with peripheral sensory neuropathy and cerebellar symptoms as part of paraneoplastic neurological process associated with superficial transitional cell carcinoma (TCC) of the bladder. We outline the difficulties and significance of reaching the correct diagnosis, and the importance of multidisciplinary team work. Thus we demonstrate the importance of maintaining an open mind to additional common and rare diagnoses and to look for rare associations particularly in patients with paraneoplastic syndrome. 

## 2. Case Report 

A 76-year-old lady presented to the outpatient clinic of the Department of Medicine for the Elderly, Worthing Hospital, UK, in November 2010. She was complaining of a three-years history of progressive leg and hand numbness and leg weakness. She had a past medical history of osteoporosis, right-sided ankle fracture, hypertension, and anxiety. She was diagnosed with depression three years ago. Her regular medications are Amlodipine 5 mg once daily, Mirtazapine 30 mg once daily, Propanolol 20 mg three times a day, and Alendronate acid 70 mg once a week. She had no significant family history, smokes 20 cigarettes a day, and is teetotal.

On physical examination at the clinic, she was haemodynamically stable. Detailed neurological examination revealed reduced power of elbow extension and finger abduction bilaterally (MRC grade 4− to 4+). All upper limb reflexes were suppressed and joint position sense was impaired to the wrists bilaterally with some impairment of pinprick sensation in gloves distribution to the level of the wrists. Lower limb examination revealed reduced power of both knee flexion and extension bilaterally (MRC grade 4) and ankle plantar flexion (grade 4). All lower limb reflexes were present but depressed, and she had downgoing plantars bilaterally. Sensation to pinprick was reduced in the lower limbs to the ankle on the right and to the midshin on the left. Joint position sense was impaired to the ankles bilaterally. She had a marked degree of truncal ataxia. She had no other neurological abnormalities. Routine blood tests showed mild neutrophilia (10.2 × 10^9^/L), the rest of her blood tests, including CRP, electrolytes, calcium, random glucose, liver function tests, INR, serum B12, folate, ferritin, and TSH, were all in the normal range. Further tests were arranged as an outpatient. Electrophysiological study revealed absent lower and upper sensory action potentials. Lower limb motor action potentials were diminished and there was mild, patching motor conducting slowing. Needle EMG showed mild-to-moderate chronic neurogenic changes. The patient has been referred to the neurology outpatient clinic with the above test results. These results clearly suggested a predominantly sensory neuropathy or neuronopathy. The degree of truncal ataxia suspected a degree of cerebellar involvement. Regarding these findings, the possibility of a paraneoplastic process has been raised. In further blood tests, autoantibody screening was ordered as well as brain imaging (MRI brain) and lumbar puncture (LP) for CSF examination to rule out inflammatory neuropathy. 

In her routine blood tests, her mild neutrophilia remained present with normal CRP and ESR results (Haemoglobin of 14.2 g/dL, white cell count 12.4 × 10^9^/L, neutrophil 8.9 × 10^9^/L, CRP <5 mg/L, ESR: 5 mm/hour). Immunology blood results showed negative antinuclear antibody (ANA) and antinuclear cytoplasmic antibody (ANCA). HIV1 and 2 and syphilis serology were also negative. Free thyroxine level (23.5 pmols/L) was just above the normal range (12–22 pmols/L) with normal TSH 0.67 mIU/L. Serum IgG level was low (3.9 g/L), and serum IgA and IgM levels were normal. Chest X-ray showed no abnormality. Urine dipstick result was in keeping with urinary infection (+ nitrate, + pus cells) without evidence of protein or haematuria. The patient's urinary tract infection was treated with nitrofurantoin due to sensitivity of the results to the coliforms present in her urine cultures. 

LP was carried out and her cerebrospinal fluid (CSF) result showed no significant abnormality (white cells 1 × 10^6^/L, Rbc negative) and there were no organisms seen. The CSF culture also came back as negative. CSF protein was raised 489.2 mg/L (normal; 150–450 mg/L), with normal CSF glucose and lactate levels. Cytology showed very occasional degenerated lymphocytes.

MRI scan of the brain showed abnormal high signal within the right medial temporal lobe associated with cortical thickening and lesser high signal in the left median temporal lobe suspicious. Unfortunately, she was not able to tolerate further studies and postcontrast images were not obtained. Western blot for Antineuronal antibody confirmed the presence of anti-HU antibody (done at Church Hill Hospital, Oxford, UK). As there was no specific symptom or sign suggesting any primary malignancy, an urgent CT chest/abdomen/pelvis with contrast was performed. It showed an 8 mm soft tissue lesion arising from the left posterior wall of the bladder. A prominent 10 mm lymph node was demonstrated within the right side of the mesorectum. 

Cystoscopy and transurethral resection of the bladder lesion has been arranged under general anaesthesia with a simultaneous, on-table flexible sigmoidoscopy. Flexible sigmoidoscopy was performed (29/06/2011) up to 22 cm from the anal verge, and no obvious pathology was seen. Rigid cystoscopy revealed a single 7–10 mm lesion arising from the left posterior wall of the bladder clinically suggesting superficial transitional cell bladder cancer (TCC) and was resected completely with a diathermy loop. Histology result of the bladder lesion came back G1 pTa TCC with focal G2 TCC areas. Postoperative intravenous ureterogram did not show any ureteric and collecting system filling defect. Follow-up surveillance cystoscopy in 3–6 months and 1 year time did not reveale any recurrence with negative urine cytology and further negative upper urinary tract imaging. The patient paraneoplastic encephalomyelitis (PEM) and subacute sensory neuronopathy (SSN) considerably improved after the resection of the tumour; however, after 1 year, she is still having neurologically detectable residual deficit. 

## 3. Discussion 

Our aim was to report the case of a female patient under our care with anti-Hu antibody mediated paraneoplastic neurological syndrome associated with transitional cell carcinoma of the bladder and to review the literature. 

It is well known that a paraneoplastic syndrome is antibody—in this case anti-Hu—mediated neurologic disorders associated with underlying tumours. The antigens expressed by the tumour are recognized by immune cells as foreign antigens, leading to the production of antibodies that recognize not only the tumour cells, but also any neural cells expressing the antigen [9]. These antibodies such as anti-Hu antibodies can be detected in patient's serum, cerebrospinal fluids by means of immunofluorescence or immunoperoxidase, confirmed by western blot or ELISA [10]. 

Before we established the diagnoses of bladder cancer with rigid cystoscopy, our patient denied any significant urological bladder symptoms such as painless macroscopic haematuria, recurrent urinary tract infection, suprapubic pain, filling-type lower urinary tract symptoms, or urgency and she had no microscopic haematuria; therefore, we would never find her cancer especially in this early stage. The systematic investigation of her subacute paraneoplastic encephalomyelitis (PEM) is a multifocal inflammatory disorder accompanied by subacute sensory neuronopathy (SSN) due to involvement of the dorsal root ganglia leading to remote neoplasia. Detection of paraneoplastic syndrome antibodies like anti-Hu should trigger a systematic search for underlying tumour just like what we demonstrated in this case report. 

Recognition of the variable manifestations of paraneoplastic neuropathy is important, as diagnosis at an earlier stage facilitates prompt treatment and provides better chances of good outcomes [11]. To our knowledge, this is so far the first case report of anti-Hu positive sensory neuropathy associated with superficial TCC of the bladder. Further study is needed to clarify the specificity and the sensitivity of ANNA-1 antibody to paraneoplastic neurological syndrome and bladder cancer. 
